# Interpretations and comments for expert consensus on the diagnosis and treatment of heat stroke in China

**DOI:** 10.1186/s40779-020-00266-4

**Published:** 2020-08-06

**Authors:** Shu-Yuan Liu, Qian Wang, Yun-Peng Lou, Yan Gao, Bo Ning, Qing Song, Hai-Ling Li

**Affiliations:** 1 Medical School of Chinese PLA, No. 28 of Fuxing Road, Beijing, 100853 China; 2 Emergency Department, Sixth Medical Center, Chinese PLA General Hospital, No. 6 of Fucheng Road, Beijing, 100048 China; 3 Emergency Department, Third Medical Center, Chinese PLA General Hospital, No. 69 of Yongding Road, Beijing, 100039 China; 4 Department of Intensive Care Unit, No. 971 Hospital of the People’s Liberation Army Navy, No. 22 Minjiang Road, Qingdao, 266071 China; 5 Emergency Department, General Hospital of Northern Theater Command of Chinese PLA, No. 83 of Wenhua Road, Shenyang, 110016 China; 6 Department of Intensive Care Unit, Air Force Medical Center of China, No.30 of Fucheng Road, Beijing, 100142 China

**Keywords:** Heat stroke, Definition, Diagnosis, Treatment, Targeted temperature management

## Abstract

Heat stroke is the most severe type of heat illness, it is often accompanied by severe multiorgan damage and has a high fatality rate. In January 2020, based on new research evidence and the experiences of Chinese experts in heat stroke, the Expert Group of Heat Stroke Prevention and Treatment of the Chinese People’s Liberation Army (PLA) and the Professional Committee of Critical Care Medicine of the Chinese PLA jointly issued a new *Expert Consensus on the Diagnosis and Treatment of Heat Stroke in China*. This article aims to interpret and supplement the major updates to the new consensus.

Heat-related injury can cause a series of mild to severe pathophysiological changes, collectively referred to as heat illness [1]. Heat stroke, often accompanied by severe multiorgan damage, is the most severe type of heat illness and has high fatality rate. In January 2020, based on new research evidence and the experiences of Chinese experts in heat stroke, the Expert Group of Heat Stroke Prevention and Treatment of the Chinese PLA and the Professional Committee of Critical Care Medicine of the Chinese PLA jointly issued a new *Expert Consensus on the Diagnosis and Treatment of Heat Stroke in China* [2] (hereinafter referred to as the “new consensus”), in which the definition of heat stroke and diagnostic criteria were updated.

To date, there is no perfect definition of heat stroke. The most commonly used definition worldwide was Bouchama’s definition that was proposed in 2002 [3]; this old definition served as a basis for the new definition of heat stroke posited in the new consensus. Although the onset of heat stroke is often associated with environmental factors (such as high temperature), this factor is not actually necessary. Some individuals may also develop heat stroke while exercising vigorously in an environment that is not too hot. In fact, the main mechanism of heat stroke is the imbalance between body heat production and dissipation, resulting in a large amount of heat accumulation in the body beyond endurance capacity, which causes extensive damage. A pathophysiological description added to the new definition makes it more consistent with the nature of heat stroke (see attachment 1 for details).

Contrary to the definition, the diagnostic criteria for heat stroke have rarely been clearly described in previous guidelines. Hyperthermia is the most prominent characteristic of heat stroke, and the core temperature is often used to distinguish the severity. However, in clinical practice, it was often found that some patients had significant manifestations of heat stroke when their measured temperature did not reach 40 °C [4–6]. In consideration of this situation, the Japanese Association for Acute Medicine (JAAM) proposed new diagnostic criteria (modified JAAM criteria) in 2016 [4], in which body temperature was not included in the diagnostic criteria. In addition, not all individuals with a body temperature of more than 40 °C experience heat stroke. Based on these considerations and the current situation of treatment in China, the new consensus proposed new diagnostic criteria consisting of two aspects, “medical history information” and “clinical manifestations”. Heat stroke should be considered if the patient meets any of the medical history information criteria while also having any of the clinical presentations (if the symptoms cannot be explained by other reasons). The new consensus no longer treats core temperature as a necessary condition for clinical diagnosis, thus avoiding delays in treatment due to “diagnostic” problems (see attachment 2 for details).

To further standardize and emphasize cooling treatment, the new consensus introduced the concept of targeted temperature management (TTM). The accurate management of body temperature might be particularly important for heat stroke. It is important to choose the most effective way to control the body temperature according to the condition of the scene. It should be emphasized that any cooling method or combination of two or more technologies should be used until more effective cooling measures are achieved. However, there is still no strong evidence to determine the optimal target temperature for cooling therapy. Most studies recommend a cooling endpoint between 38.0 °C and 39.0 °C. It should be noted that these recommendations were based only on the results observed in the field treatment of athletes and lacked strong scientific evidence. In theory, mild hypothermia does not occur until the core temperature drops to 35.0 °C, so systemic cooling in patients with heat stroke can be continued until clinical improvement or rectal temperature ranges from 37.0 °C to 38.0 °C as opposed to depending on a certain temperature node. Combined with the above evidence and debates, the new consensus suggests that the goal of core temperature management is to maintain the rectal temperature at 37.0 °C to 38.5 °C, which is a broad range (see attachment 3 for details).

## Supplementary information

**Additional file 1.** Updated definition of heat stroke.

**Additional file 2.** Updated diagnostic criteria for heat stroke.

**Additional file 3.** Targeted temperature management in patients with heat stroke.

## Data Availability

The datasets used and/or analyzed are available from the corresponding authors on reasonable request.

## Abbreviations

JAAM: Japanese Association for Acute Medicine
TTM: Targeted temperature management
