# Supplementary material for: Interpretations and comments for expert consensus on the diagnosis and treatment of heat stroke in China
Source: Mil Med Res. 2020 Aug 6;7:37. doi: 10.1186/s40779-020-00266-4 (PMC7412797; doi:10.1186/s40779-020-00266-4)
Supplement: Supplementary file 1 — Additional file 1. Updated definition of heat stroke. [file 40779_2020_266_MOESM1_ESM.docx]

# Attachment 1

# Updated definition of heat stroke

To date, there is no perfect definition of heat stroke. The most commonly used definition worldwide was Bouchama’s definition proposed edin 2002 [1], that is, a core temperature rising above 40℃ accompanied by dry skin and central nervous system abnormalities, such as delirium, convulsions or coma. Exposure to high temperatures or strenuous exercises could cause heat stroke. Most subsequent studies or guidelines have referenced this definition but lacked a clear description [2-5], and some studies have not specified whether the term “body temperature” refers to the core temperature [6].

Bouchama’s definition had three basic elements: (1) exposure to the thermal environment, (2) core temperature > 40 ℃, and (3) central nervous system abnormalities. The new consensus put forward a new definition of heat stroke, which was supplemented and improved on the basis of Bouchama’s definition: a life-threatening clinical syndrome of imbalance between heat production by and dissipation from the body caused by exposure to a hot environment and/or strenuous exercises, characterized by a core temperature of > 40 ℃ and abnormalities of the central nervous system, including changes in mental status, convulsions or coma and accompanied by multiple organ damage.

A description of the pathophysiology was added in the new definition. Although the onset of heat stroke is often associated with environmental factors (such as high temperature), this factor is not actually necessary. For example, if the ambient temperature is not too high, but the humidity is high, ventilation is poor, or the clothing is thick, which affects heat dissipation, heat stroke can also occur. Some individuals may also develop heat stroke while exercising vigorously in an environment that is not too hot. It was reported that a 34-year-old man developed heat stroke during a half marathon race. The day was sunny, and the ambient temperature was 24.2 ℃ [7]. In fact, the main mechanism of heat stroke is the imbalance between body heat production and dissipation, resulting in a large amount of heat accumulation in the body beyond endurance capacity, which causes extensive damage. A pathophysiological description added to the new definition makes it more consistent with the nature of heat stroke.

As a matter of fact, there are indeed clinical cases of heat stroke with a body temperature that is not too high (< 40 ℃) [8]. A 42-year-old man in America was convulsive and unconscious in a closed car; the patient’s symptoms were accompanied by disseminated intravascular coagulation (DIC) and multiple organ failure. Although the patient’s core temperature measured by urinary catheter was 38.8 ℃, he was diagnosed with heat stroke [9]. The use of rectal temperatures is now the "gold standard" for assessing core temperature and is recommended by the National Athletic Trainers Association Position Statement on Exertional Heat Illness. Indeed, it is often difficult to obtain accurate body temperature on the first attempt due to the interference of various factors, such as individual acclimatized status, measurement timing (e.g., cooling before measurement), inaccurate temperature reading and measurement site, which can all lead to diverse results. In addition, the true thermal indicator of tissue and organ damage is core temperature rather than body surface temperature (such as axillary temperature). However, core temperature measurement at the scene of onset is often subject to many conditions. The experts of consensus therefore diverged on whether to retain “body temperature” when discussing the new definition. However, define and diagnose one disease is different. Define a disease need characteristics of clinical presentation whereas diagnosis criteria reflect different features of the disease from others. The definition should reflect the most essential characteristics of diseases, while the diagnostic criteria should facilitate the identification and differentiation of diseases. Heat stroke is a serious heat-related disease in nature, and core temperature is still the most direct indicator of “heat”. If the measured body temperature was not too high in some patients with heat stroke, it does not mean that their actual core temperature is not high because there are many uncertain factors influencing the measured values. After discussion, the panel therefore determined that the term “body temperature” should remain in the new definition. However, it is still unclear what the appropriate threshold core temperature for heat stroke is, and the available evidence has indicated that the core temperature in most patients with heat stroke is much higher than 40 ℃. Therefore, the new consensus still used “core temperature >40 ℃” in the new definition.

# References

1. Bouchama A, Knochel JP. Heat stroke. N Engl J Med. 2002; 346(25): 1978-88.
2. Lipman GS, Eifling KP, Ellis MA, Gaudio FG, Otten EM, Grissom CK, et al. Wilderness Medical Society practice guidelines for the prevention and treatment of heat-related illness: 2014 update. Wilderness Environ Med. 2014; 25(4 Suppl): S55-65.
3. Casa DJ, DeMartini JK, Bergeron MF, Csillan D, Eichner ER, Lopez RM, et al. National Athletic Trainers' Association Position Statement: Exertional Heat Illnesses. J Athl Train. 2015; 50(9): 986-1000.
4. Belval LN, Casa DJ, Adams WM, Chiampas GT, Holschen JC, Hosokawa Y, et al. Consensus Statement- Prehospital Care of Exertional Heat Stroke. Prehosp Emerg Care. 2018; 22(3): 392-397.
5. Lipman GS, Gaudio FG, Eifling KP, Ellis MA, Otten EM, Grissom CK. Wilderness Medical Society Clinical Practice Guidelines for the Prevention and Treatment of Heat Illness: 2019 Update. Wilderness Environ Med. 2019; 30(4S): S33-S46.
6. Misset B, De Jonghe B, Bastuji-Garin S, Gattolliat O, Boughrara E, Annane D, et al. Mortality of patients with heatstroke admitted to intensive care units during the 2003 heat wave in France: a national multiple-center risk-factor study. Crit Care Med. 2006; 34(4): 1087-92.
7. Yoshizawa T, Omori K, Takeuchi I, Miyoshi Y, Kido H, Takahashi E, et al. Heat stroke with bimodal rhabdomyolysis: a case report and review of the literature. J Intensive Care. 2016; 4:71.
8. Hifumi T, Kondo Y, Shimazaki J, Oda Y, Shiraishi S, Wakasugi M, et al. Prognostic significance of disseminated intravascular coagulation in patients with heat stroke in a nationwide registry. J Crit Care. 2018; 44: 306-311.
9. Knoll JM, Knight LR, Quiroz D, Popat SM, Pederson TG, Morton-Gonzaba N. Variation in Clinical Presentations and Outcomes of Heat Stroke Victims in the Mass-Casualty Setting. J Emerg Med. 2019; 57(6): 866-870.
