# Supplementary material for: Interpretations and comments for expert consensus on the diagnosis and treatment of heat stroke in China
Source: Mil Med Res. 2020 Aug 6;7:37. doi: 10.1186/s40779-020-00266-4 (PMC7412797; doi:10.1186/s40779-020-00266-4)
Supplement: Supplementary file 2 — Additional file 2. Updated diagnostic criteria for heat stroke. [file 40779_2020_266_MOESM2_ESM.docx]

# Attachment 2

# Updated diagnostic criteria for heat stroke

Contrary to the definition, the diagnostic criteria for heat stroke have rarely been clearly described in previous guidelines. When Bouchama and Knochel proposed the definition of heat stroke in 2002, they unfortunately did not provide a clear diagnostic criterion. However, they suggested that the diagnosis of heat stroke must include two manifestations [1]: the first was hyperthermia, often with a core temperature range of 40 ~ 47 ℃, and the second was central nervous system dysfunction, which was usually severe but could be mild, characterized only by inappropriate behavior or impaired judgment, but more commonly delirium or frank coma. Most subsequent guidelines or studies have adopted this recommendation, especially since core temperature > 40 ℃ has become essential for the diagnosis of heat stroke [2-5].

Hyperthermia is the most prominent characteristic of heat stroke, and the core temperature is often used to distinguish the severity. However, in clinical practice, it was often found that some patients had significant manifestations of heat stroke when their measured temperature did not reach 40 ℃. In consideration of this situation, the Japanese Association for Acute Medicine (JAAM) proposed new diagnostic criteria (modified JAAM criteria) in 2016, which included exposure to high environmental temperature and meeting at least one of the following criteria: (1) Glasgow Coma Scale (GCS) score of ≤14, (2) creatinine or total bilirubin levels of ≥1.2 mg/dL, and (3) JAAM DIC score ≥ 4. Notably, body temperature was not included in the diagnostic criteria.

Actually, not all individuals with a body temperature of more than 40 ℃ experience heat stroke. An earlier study measured the body temperature of two athletes during a marathon. The rectal temperatures were as high as 41.9 ℃, but both of them finished the race well without any manifestations of heat stroke [6]. A recent field study using orally ingested capsules to measure the core temperature showed that the intestinal temperature of road cycling athletes in hot weather continued to reach 41.5 ℃ without any signs of heat stroke [7]. Therefore, the highest body temperature in patients with heat stroke may have a poor correlation with the degree of organ damage, and the core temperature may lack satisfactory sensitivity and specificity for both diagnosis and severity evaluation. In addition, the measurement of core temperature at the onset site is often conditioned, while the measurement of body surface temperature has indicated a significant number of patients < 40 ℃, which may lead to the delay of diagnosis if temperature is included in the necessary conditions for diagnosis.

Based on the above considerations and the current situation of treatment in China, the new consensus proposed new diagnostic criteria consisting of two aspects, “medical history information” and “clinical manifestations”. Medical history information: (1) patients exposed to high temperature and high humidity environments; and (2) patients subjected to high intensity exercise. Clinical manifestations: (1) functional impairment of the central nervous system (such as coma, convulsions, delirium and abnormal behavior); (2) core temperature exceeding 40 ℃; (3) functional impairment of multiple organs (≥2) (such as liver, kidney, striated muscle and gastrointestinal tract); and (4) severe coagulopathy or DIC. Heat stroke should be considered if the patient meets any of the medical history information criteria while also having any of the clinical presentations (if the symptoms cannot be explained by other reasons). The new consensus no longer treats core temperature as a necessary condition for clinical diagnosis to avoid delays in treatment due to “diagnostic” problems. If the patient is consistent from history to clinical presentation, heat stroke cannot be excluded simply and arbitrarily because the body temperature (including core temperature) does not exceed 40 ℃.

# References

1. Bouchama A, Knochel JP. Heat stroke. N Engl J Med. 2002; 346(25): 1978-88.
2. Lipman GS, Eifling KP, Ellis MA, Gaudio FG, Otten EM, Grissom CK, et al. Wilderness Medical Society practice guidelines for the prevention and treatment of heat-related illness: 2014 update. Wilderness Environ Med. 2014; 25(4 Suppl): S55-65.
3. Casa DJ, DeMartini JK, Bergeron MF, Csillan D, Eichner ER, Lopez RM, et al. National Athletic Trainers' Association Position Statement: Exertional Heat Illnesses. J Athl Train. 2015; 50(9): 986-1000.
4. Belval LN, Casa DJ, Adams WM, Chiampas GT, Holschen JC, Hosokawa Y, et al. Consensus Statement- Prehospital Care of Exertional Heat Stroke. Prehosp Emerg Care. 2018; 22(3): 392-397.
5. Lipman GS, Gaudio FG, Eifling KP, Ellis MA, Otten EM, Grissom CK. Wilderness Medical Society Clinical Practice Guidelines for the Prevention and Treatment of Heat Illness: 2019 Update. Wilderness Environ Med. 2019; 30(4S): S33-S46.
6. Maron MB, Wagner JA, Horvath SM. Thermoregulatory responses during competitive marathon running. J Appl Physiol Respir Environ Exerc Physiol. 1977; 42(6): 909-14.
7. Racinais S, Moussay S, Nichols D, Travers G, Belfekih T, Schumacher YO, et al. Core temperature up to 41.5ºC during the UCI Road Cycling World Championships in the heat. Br J Sports Med. 2019; 53(7): 426-429.
