# Supplementary material for: Interpretations and comments for expert consensus on the diagnosis and treatment of heat stroke in China
Source: Mil Med Res. 2020 Aug 6;7:37. doi: 10.1186/s40779-020-00266-4 (PMC7412797; doi:10.1186/s40779-020-00266-4)
Supplement: Supplementary file 3 — Additional file 3. Targeted temperature management in patients with heat stroke. [file 40779_2020_266_MOESM3_ESM.docx]

# Attachment 3

# Targeted temperature management in patients with heat stroke

To further standardize and emphasize cooling treatment, the new consensus introduced the concept of targeted temperature management (TTM). TTM is a therapeutic strategy to achieve and maintain specific core temperatures in specific patient populations to improve clinical outcomes [1]. Accurate management of body temperature might be particularly important for heat stroke, requiring continuous TTM throughout treatment. If possible, all patients with heat stroke need to have their core temperature measured immediately. If the core temperature is higher than the target temperature, continuous cooling should be performed. If the core temperature has reached the target temperature, the body temperature should be continuously monitored to avoid hypothermia or rewarming. What needs to be emphasized here is the core temperature rather than body surface temperature, and commonly used measurements of the ear canal, eardrum, mouth, armpit, and temporal regions do not represent core temperature well and may be misleading.

We should choose the most effective way to control the body temperature according to the condition of the scene. Among cooling techniques, cold water immersion (CWI) is by far the most effective. Intravascular cooling may also be an effective method if available, but it requires special equipment, is technically demanding, and has been less reported. Cooling methods such as wet towels, arterial ice compresses, fans or sprays are less effective. However, it should be emphasized that any cooling method or combination of two or more technologies should be used until more effective cooling measures are achieved. The basic principle of early management at onset site is “transfer while cooling”. When there is a conflict between cooling and transport, the principle of “cooling first, transfer second” should be followed [2-5]. Because of the dysfunction of the temperature regulation center in the early onset of heat stroke, it is not recommended to use drugs for cooling, such as aspirin, indomethacin, and artificial hibernation mixtures.

There is still no strong evidence to determine the optimal target temperature for cooling therapy. Most studies recommend a cooling endpoint between 38.0 and 39.0 ℃ [2, 6-9]. However, these recommendations were based only on the results observed in the field treatment of athletes and lacked strong scientific evidence. In theory, mild hypothermia does not occur until the core temperature drops to 35 ℃, so systemic cooling in patients with heat stroke can be continued until clinical improvement or rectal temperature ranges from 37 ℃ to 38 ℃ as opposed to depending on a certain temperature node. Combined with the above evidence and debates, the new consensus suggested that the goal of core temperature management is to maintain rectal temperature at 37.0 ℃ to 38.5 ℃, which is a broad range. If the temperature rises again, it is recommended to restart cooling measures. It should be emphasized that the patient’s performance should be closely observed during the cooling process, especially neurological manifestations such as consciousness, behavior, and cognitive function.

# References

1. Stanger D, Mihajlovic V, Singer J, Desai S, El-Sayegh R, Wong GC. Editor's Choice-Effects of targeted temperature management on mortality and neurological outcome: A systematic review and meta-analysis. Eur Heart J Acute Cardiovasc Care. 2018; 7(5): 467-477.
2. Casa DJ, DeMartini JK, Bergeron MF, Csillan D, Eichner ER, Lopez RM, et al. National Athletic Trainers' Association Position Statement: Exertional Heat Illnesses. J Athl Train. 2015; 50(9): 986-1000.
3. Belval LN, Casa DJ, Adams WM, Chiampas GT, Holschen JC, Hosokawa Y, et al. Consensus Statement- Prehospital Care of Exertional Heat Stroke. Prehosp Emerg Care. 2018; 22(3): 392-397.
4. Pryor RR, Casa DJ, Holschen JC, O'Connor FG, Vandermark LW. Exertional heat stroke: strategies for prevention and treatment from the sports field to the emergency department. Clin Pediatr Emerg Med. 2013; 14(4): 267-278.
5. Sloan BK, Kraft EM, Clark D, Schmeissing SW, Byrne BC, Rusyniak DE. On-site treatment of exertional heat stroke. Am J Sports Med. 2015; 43(4): 823-829.
6. Gagnon D, Lemire BB, Casa DJ, Kenny GP. Cold-water immersion and the treatment of hyperthermia: using 38.6℃ as a safe rectal temperature cooling limit. J Athl Train. 2010; 45(5): 439-444.
7. Walter E, Steel K. Management of exertional heat stroke: a practical update for primary care physicians. Br J Gen Pract. 2018; 68(668): 153-154.
8. Nicole W. Mitigating Climate Impacts on Athletes: Sports Guidelines May Prevent Exertional Heat Illness. Environ Health Perspect. 2019; 127(10): 104001.
9. Alzeer AH, Wissler EH. Theoretical analysis of evaporative cooling of classic heat stroke patients. Int J Biometeorol. 2018; 62(9): 1567-1574.
